# Anti-obesity effect of a traditional Chinese dietary habit—blending lard with vegetable oil while cooking

**DOI:** 10.1038/s41598-017-14704-2

**Published:** 2017-10-31

**Authors:** Ji Wang, Sisi Yan, Haisi Xiao, Huijuan Zhou, Shuiping Liu, Yu Zeng, Biying Liu, Rongfang Li, Zhihang Yuan, Jing Wu, Jine Yi, Yarou Bao Sero Razack, Lixin Wen

**Affiliations:** 1 Laboratory of Animal Clinical Toxicology, Department of Clinical Veterinary Medicine, College of Veterinary Medicine, Hunan Agricultural University, Changsha, Hunan Province P.R. China; 2 Hunan Collaborative Innovation Center of Animal Production Safety, Changsha, Hunan Province P.R. China

## Abstract

Obesity, which is associated with dietary habits, has become a global social problem and causes many metabolic diseases. In China, both percentages of adult obesity and overweight are far lower compared to western countries. It was designed to increase the two levels of daily intake in human, namely 3.8% and 6.5%, which are recommendatory intake (25 g/d) and Chinese citizens’ practical intake (41.4 g/d), respectively. The mice were respectively fed with feeds added with soybean oil, lard or the oil blended by both for 12 weeks. In the mice fed with diet containing 3.8% of the three oils or 6.5% blended oil, their body weight, body fat rate, cross-sectional area of adipocytes, adipogenesis and lipogenesis in adipose were decreased, whereas hydrolysis of triglyserides in adipose was increased. This study demonstrated that the oil mixture containing lard and soybean oil had a remarkable anti-obesity effect. It suggests that the traditional Chinese dietary habits using oils blended with lard and soybean oil, might be one of the factors of lower percentages of overweight and obesity in China, and that the increasing of dietary oil intake and the changing of its component resulted in the increasing of obesity rate in China over the past decades.

## Introduction

The rise in obesity has aroused increasing concerns around the world. In recent decades, the obesity prevalence rates are enhancing with rapid speed far beyond expectations, as evidenced by the fact that in 2014, about 266 million men and 375 million women were found to be obese in the world, compared to 34 million men and 71 million women in 1975. Among different countries, China has more obese men and women than the USA, becoming the largest obese population^1^. Notably, obesity can lead to huge personal, social, and economic costs. McKinsey Global Institute has reported that obesity results in about 5% of global death, and the global economic cost from obesity is close to $2.0 trillion, around 2.8% of global GDP^2^.

The development of obesity is obviously relevant to genetic and epigenetic factors including dietary habits. Interestingly, as the 2016 *Global Nutrition Report* showed, the dietary habits in western countries are characterized by high fat, carbohydrate and calorie, and the adult obesity prevalence in most of the western countries, especially in the USA and the western of Europe is approximately 30%, while the adult overweight prevalence has already exceeded 60%. In countries that familiarized to Mediterranean diets, which are commonly acknowledged as a healthy dietary habit, the prevalence of adult obesity is over 20%, and the prevalence of adult overweight are around 60%. In China, the percentage of adult obesity and overweight are 6.9% and 34.4%, respectively, and notably, both are far below western countries^3^. Lard is one of the most popular cooking-oils in Chinese gastronomy, and it has great effect on diuresis, blood clearance, activating blood circulation, and detoxification. It has been reported that people with longevous lifespan in China were used to eat pork braised in brown sauce, a Chinese food rich in blend oil.

From the perspective of concentration of fatty acids, the contents of saturated fatty acids (SFAs) or monounsaturated fatty acids (MUFAs) are considerably higher than polyunsaturated fatty acids (PUFAs) in lard, whereas in majority of vegetable oils, it contain the higher level of PUFAs or MUFAs but the lower level of SFAs than lard. It has been proposed that dietary MUFAs and PUFAs are correlated with reduction of coronary heart disease (CHD)^4–6^, cardiovascular disease (CVD), hypertension, diabetes mellitus, anorexia nervosa, and obesity^7,8^. Increasing intake of a high SFAs diet can also be a risky factor for hypertension^9^, hypercholesterolemia^10^ and CVD^11^. However, these results are controverted by other studies^12,13^. SFAs play crucial roles in normal cellular and tissue metabolism and they function as the same importance as MUFAs and PUFAs. For instance, it has been suggested that MUFAs might not be the proper substitute for SFAs^14^, and replacement dietary SFAs with ω-6 linoleic acid, shows no evidence of cardio-protection^15^. In addition, SFAs reduction alone is not enough to reduce the risk ofCHD^10^.

In healthy people, the majority of fatty acids consumed in the diet are accessible to the bloodstream through efficient process of digestion and absorption^16^. Whether a qualitative or a quantitative imbalance of dietary fatty acids has been implicated in a number of chronic diseases as mentioned above. The World Health Organization (WHO) and China’s Public Union of Nutrition recommend that the proportion of calorie from lipid should not be lower than 20% or higher than 30% in adult daily food, and the ratio of SFAs, MUFAs and PUFAs should be preferably 1:1:1. Among the Chinese gastronomy habits, many Chinese people prefer to blend lard with vegetable oil or use them as an alternation while cooking; in this way, they would not only advance the tastes of food, but also reduce the consumption of total oil by one third. From the point of fatty acids, the ratio of SFAs: MUFAs: PUFAs is closer to 1: 1: 1 through this method than using simple oil.

During the past few decades, it has been dramatically changed in Chinese dietary pattern. According to the data from *Nutrition and Health Status of Chinese Residents*, pressed by State Council Information Office of China in 2004, it has been reported that the consumption of cooking oil was increased to 41.4 g per day, which is far beyond the recommendation that should be taken per day (25~30 g cooking oil). In addition, there is an imbalance in the usage of daily cuisine oil, and an increasing number of people tend to choose vegetable oil rather than lard. The daily consumption of oil contains 31.7 g vegetable oils and 8.7 g animal fats, and the proportion of soybean oil is more than 40% among the cooking vegetable oils. Correspondingly, the morbidities of metabolic diseases, e.g. obesity, hypertension, and type 2 diabetes have been increased as well. It may be implicated that these diseases and metabolic disorders are closely correlated with the oil intake pattern. In view of these points, this study was aimed to investigate the effect of Chinese oil consumption habit on fat deposition in mouse models. It was designed to enhance two levels of daily intake in human, which are recommendatory volume (25 g/d) and Chinese citizens’ practical volume (41.4 g/d), amount to 3.8% and 6.5% oil of diet in mice model respectively. The mice were fed with different types of oils, including soybean oil, lard and the two oil mixtures, for 12 weeks. We revealed the correlation between obesity and traditional Chinese oil intake custom, and unraveled the mysteries of lower prevalence of obesity in Chinese.

## Results

### Body weight and blood lipids

Soybean oil led to a significant increment of final body weight of mice fed with diet containing 6.5% oil (*P* < 0.01), and a decreased trend of final body weight of mice in BO-H (6.5% blended oil) group was noticed (*P* > 0.05) (Table 1). We found that serum lipid parameters of mice were also changed by oil consumption. Serum TG level of mice in SO-H (6.5% soybean oil) group and LO-H (6.5% lard oil) group were significantly higher than SO-L (3.8% soybean oil) group and LO-L (3.8% lard oil) group, respectively (*P* < 0.01). Serum TG level in BO-H group were the lowest among the 6.5% oil groups (*P* < 0.01) (Table 1). Blended oil significantly increased level of high density lipoprotein cholesterol (HDL-C) compared to other groups both in 3.8% oil groups and 6.5% oil groups (*P* < 0.05) (Table 1). Blended oil remarkably enhanced the content of low density lipoprotein cholesterol (LDL-C) in mice fed with diet of 6.5% oil compared to other groups (*P* < 0.01) (Table 1). In addition, HDL-C/ LDL-C in serum of mice in BO-L (3.8% blended oil) group markedly higher than SO-L group (*P* < 0.05), LO-L group (*P* < 0.01), and BO-H group (*P* < 0.01) (Table 1).

**Table 1:** Characteristics of weight and lipid in serum and adipose in mice fed with different oils. Abbreviations: TG, triglyceride; HDL-C, high density lipoprotein-cholesterol; LDL-C, low density lipoprotein-cholesterol. Results were presented as means ± s.e.m. Comparisons between groups were performed using one-way ANOVA with the LSD post hoc analysis (n = 10). ^#^ *P* < 0.05, ^##^ *P* < 0.01 compared with congener oil at 3.8% oil group. Different lowercase superscripts (a-c) indicate significant differences among the same oil intake level groups in the same row at *P* < 0.05 level, different lowercase superscripts (A–C) indicate extremely significant differences among the same oil intake level groups in the same row at *P* < 0.01 level.

Parameters	3.8% oil			6.5% oil		
	Soybean oil	Lard oil	Blended oil	Soybean oil	Lard oil	Blended oil
Initial body weight(g)	25.02 ± 0.49	24.59 ± 0.39	24.33 ± 0.61	24.86 ± 0.49	24.75 ± 0.22	23.85 ± 0.39
Final body weight(g)	30.54 ± 0.65	29.70 ± 0.67	28.57 ± 0.63	32.67 ± 1.08^B#^	29.70 ± 0.67 ^A^	28.80 ± 0.73 ^A^
Serum TG (mmol/l)	0.44 ± 0.01	0.41 ± 0.03	0.47 ± 0.03	0.42 ± 0.04	0.40 ± 0.02^a^	0.49 ± 0.02^b^
Serum HDL-C (mmol/l)	1.98 ± 0.07^a^	1.94 ± 0.06^a^	2.22 ± 0.09^b^	2.04 ± 0.07^a^	1.98 ± 0.06 ^A^	2.31 ± 0.11^bB^
Serum LDL-C (mmol/l)	0.16 ± 0.01	0.16 ± 0.01	0.15 ± 0.01	0.19 ± 0.01 ^A#^	0.18 ± 0.01 ^A^	0.22 ± 0.01B^##^
HDL-C/ LDL-C in serum	12.85 ± 0.46^a^	12.27 ± 0.59 ^A^	14.77 ± 0.89^bB^	11.28 ± 0.72	11.20 ± 0.37	10.65 ± 0.75^##^

### Lipids accumulation in fat tissue

**Figure 1:**
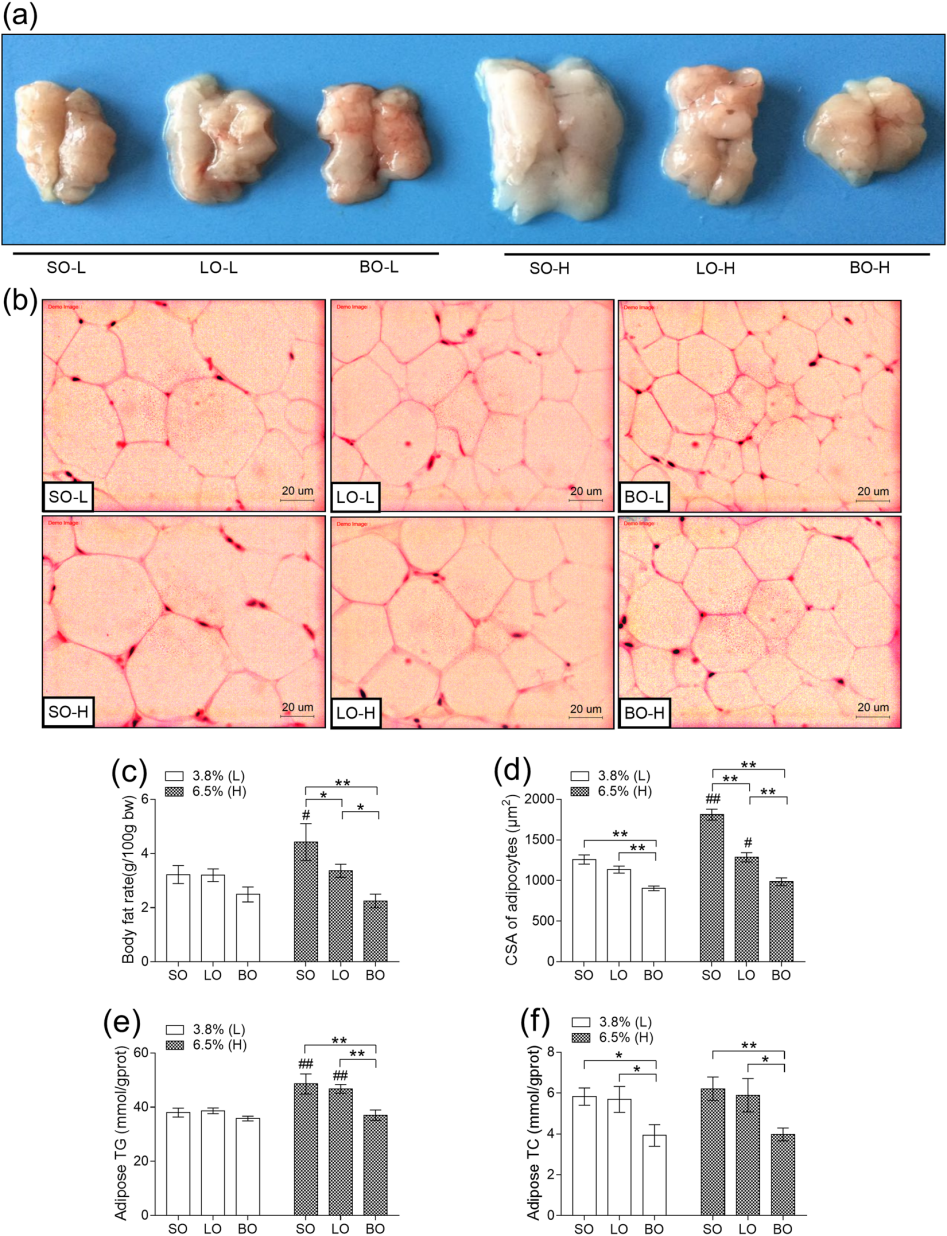
Effect of different types of oils and contents on adipose. (**a**) Epididymal adipose tissue; (**b**) Sections of epididymal adipose tissue stained with hematoxylin and eosin (H&E); (**c**) Body fat rate; (d) CSA (cross sectional area) of epididymal adipose cells; (**e**) Adipose TG (triglyceride); (**f**) Adipose TC (total cholesterol). Results were presented as means ± s.e.m. Comparisons between groups were performed using one-way ANOVA with the LSD post hoc analysis (n = 10 in c,e and f; n=5 in d). **indicated p < 0.01 when compared with different types of oils at the same content; *indicated p < 0.05 when compared with different types of oils at the same content. ^##^Indicated p < 0.01 when compared 6.5% oil with 3.8% oil groups.

Furthermore, a significant difference was observed in the body fat rate, and BO-H was the lowest whereas SO-H was the highest in the group of diet containing 6.5% blended oil (*P* < 0.01) (Fig. 1a,c). To determine the effect of different kinds of oils on the adipose, we checked triglycerides (TG) (Fig. 1e) and total cholesterol (TC) (Fig. 1f) as well as pathological section of epididymal adipose tissues (Fig. 1b,d). The contents of TG in BO-H was significantly decreased while compared with LO-H and SO-H (*P* < 0.01). There was a significant decrement of TC in the mice fed with diet of blended oil than soybean oil and lard (*P* < 0.01). Tendency of cross-sectional area (CSA) of adipocytes was similar to TC, TG. Histological examination revealed that compared with the two single oils, supplementation with blended oil resulted in reduced CSA of adipocytes (*P* < 0.01), and conversely, significant increment of CSA of adipocytes was observed in mice fed with diet of soybean oil (*P* < 0.01). It was worth noting that higher content of oil had more obvious influence on TG and CSA of adipocytes, especially in mice fed with diet containing soybean oil. These results demonstrate that blended oil can reduce body weight and content of lipid on adipose whereas higher oil enhances the content of fat on adipose.

### Lipids accumulation in liver

**Figure 2:**
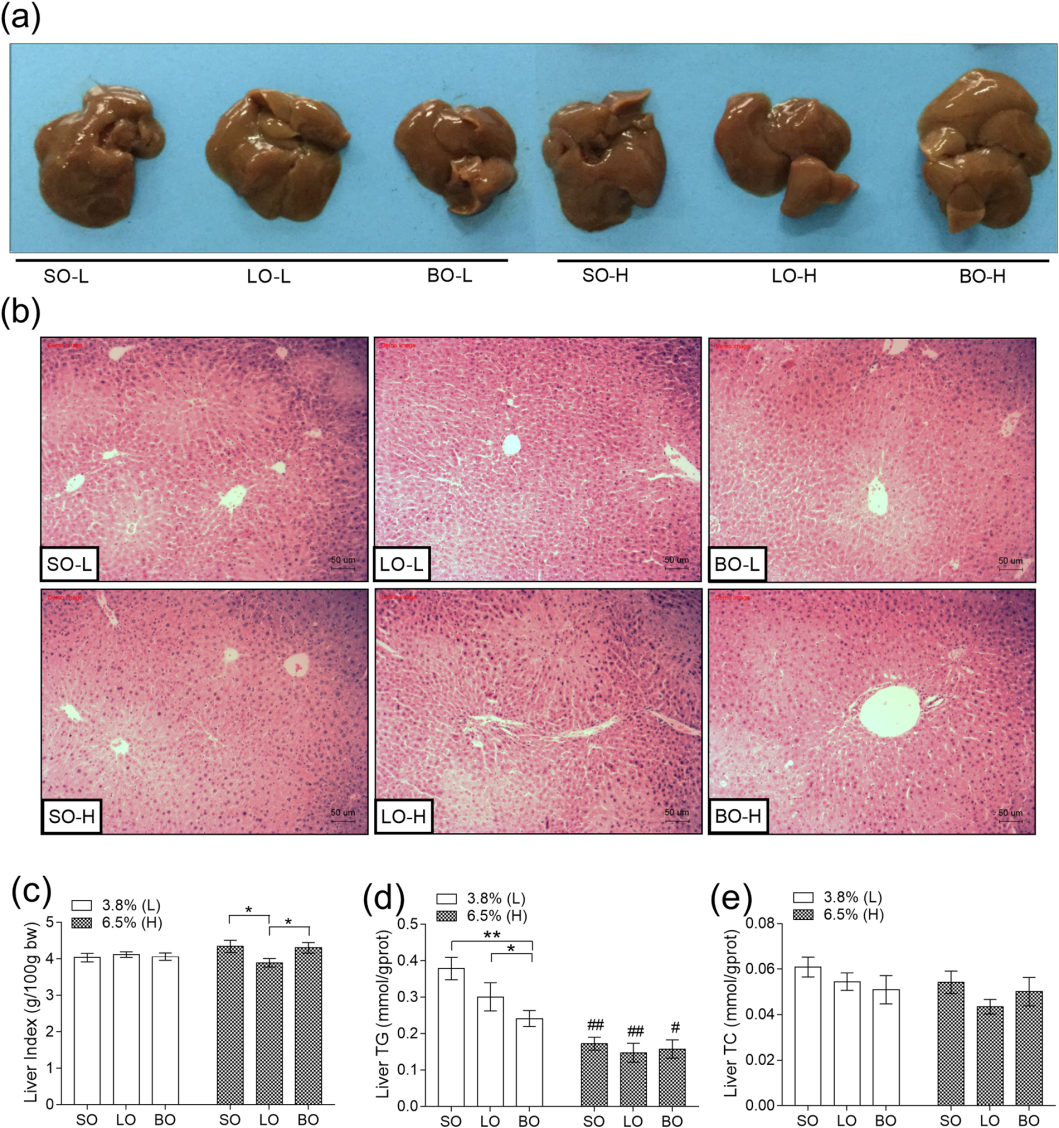
Effect of different types of oils and contents on liver. (**a**) Liver tissue; (**b**) Sections of liver stained with hematoxylin and eosin (H&E); (**c**) Liver index; (**d**) Liver TG; (**e**) Liver TC. Results were presented as means ± s.e.m. Comparisons between groups were performed using one-way ANOVA with the LSD post hoc analysis (n = 10). **indicated p < 0.01 when compared with different types of oils at the same content; *indicated p < 0.05 when compared with different types of oils at the same content. ^##^Indicated p < 0.01 when compared 6.5% oils with 3.8% oil groups. ^#^Indicated p < 0.05 when compared with 6.5% oil with 3.8% oil groups.

We next detected several indexes of liver, which indicates lipid metabolism. Liver indexes were sharply decreased in LO-H compared to SO-H and BO-H (*P* < 0.05) (Fig. 2a,c). Further, lipid in mouse liver was also tested. There was no significant change of liver TC between the groups using different types of oils (*P* > 0.05) (Fig. 2e). We observed that 6.5% oil groups assumed lower liver TG level than 3.8% oil groups (*P* < 0.05) (Fig. 2d), and histological examination no parenchymal hepatic injury (Fig. 2b).

### Expression of mRNA and protein in fat tissue

**Figure 3:**
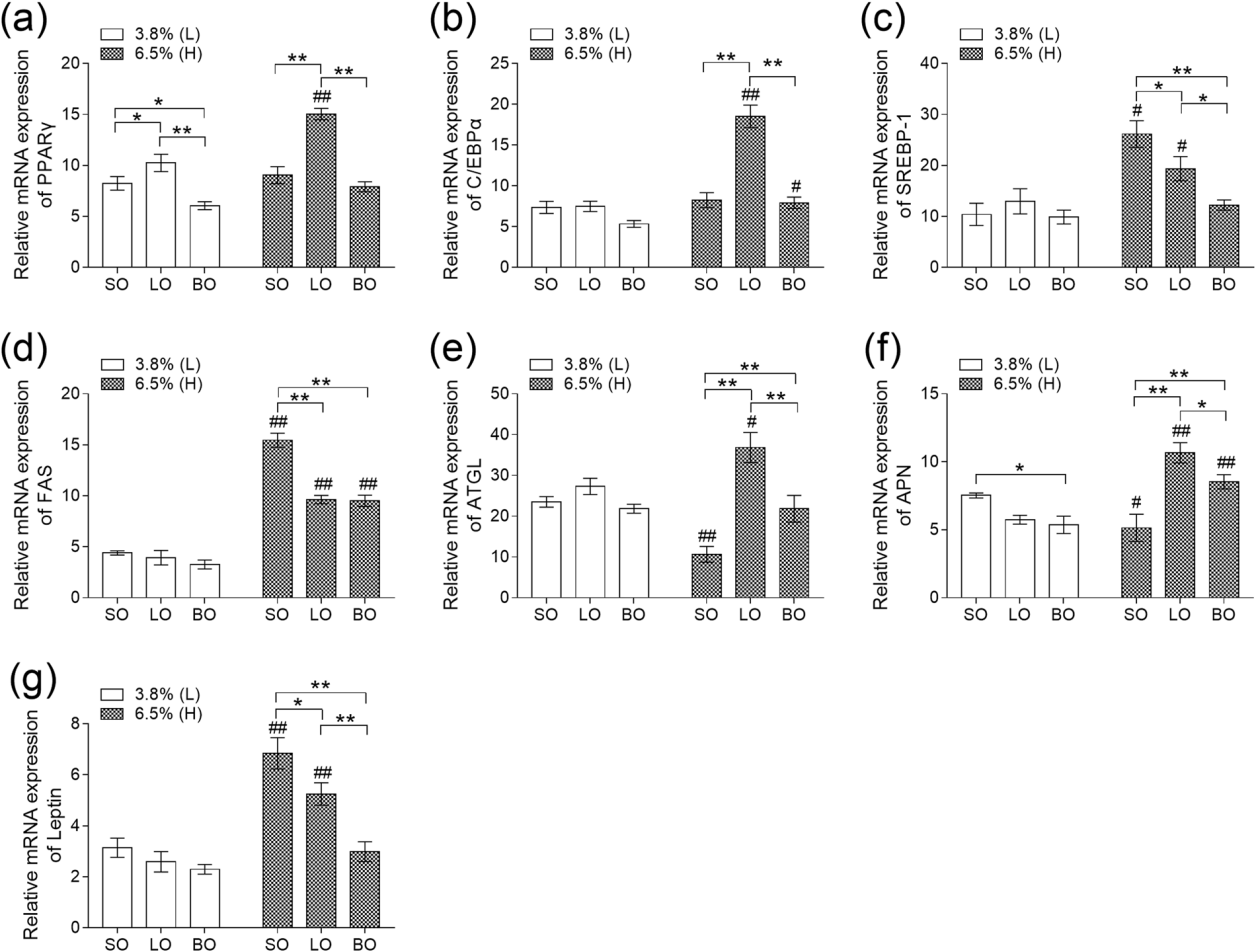
Effect of different types of oils and content on mRNA expression in epididymal adipose tissue. (**a**) Expression of PPARγ mRNA; (**b**) Expression of CEBPα mRNA; (**c**) Expression of SREBP-1 mRNA; (**d**) Expression of FAS mRNA; (**e**) Expression of ATGL mRNA; (**f**) Expression of APN mRNA; (**g**) Expression of Leptin mRNA. Results were presented as means ± s.e.m. Comparisons between groups were performed using one-way ANOVA with the LSD post hoc analysis (n = 5). **indicated p < 0.01 when compared with different types of oils at the same content; *indicated p < 0.05 when compared with different types of oils at the same content. ^##^Indicated p < 0.01 when compared 6.5% oils with 3.8% oil groups. ^#^Indicated p < 0.05 when compared with 6.5% oil with oil 3.8% oil groups.

**Figure 4:**
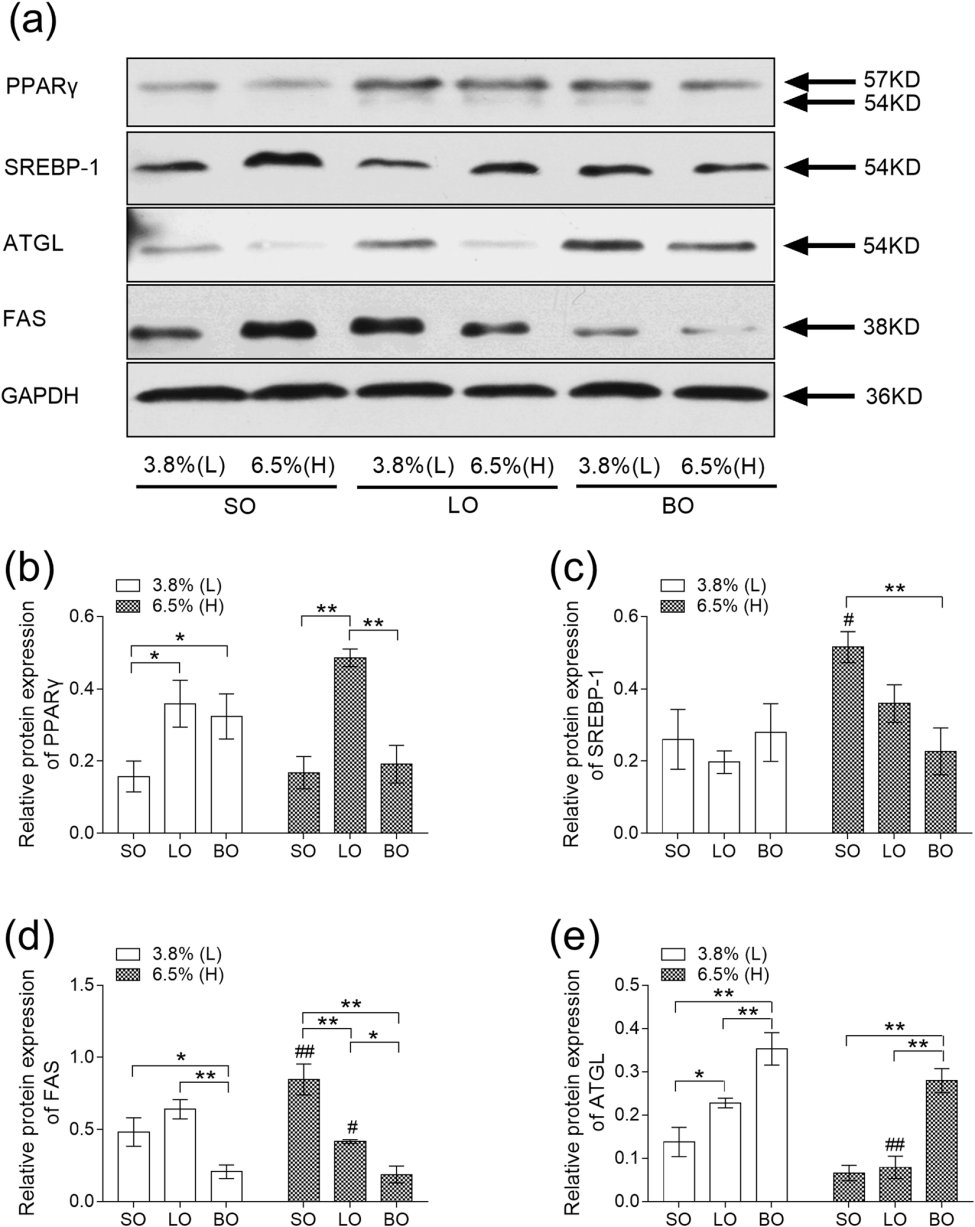
Effect of various kinds of oils and content on protein expression levels in epididymal adipose tissues. (**a**)Western blot analysis showed the levels of PPARγ, SREBP-1, ATGL, FAS, GAPDH. (**b**) Expression of PPARγ protein; (**c**) Expression of SREBP-1 protein; (**d**) Expression of FAS protein; (**e**) Expression of ATGL protein. Results were presented as means ± s.e.m. Comparisons between groups were performed using one-way ANOVA with the LSD post hoc analysis (n = 3). **indicated *P* < 0.01 when compared with different types of oils at the same content; *indicated *P* < 0.05 when compared with different types of oils at the same content. ^##^Indicated *P* < 0.01 when compared 6.5% oils with 3.8% oil groups. ^#^Indicated *P* < 0.05 when compared with 6.5% oil with oil 3.8% oil groups.

Based on the results of biochemical parameters mentioned above, we next probed the mechanism of role of blended oil. Real time PCR showed that mRNA levels of peroxisome proliferator-activated receptor γ (PPARγ) and CCAAT/enhancer- binding proteins α (C/EBPα) were higher in LO-H than SO-H and BO-H (*P* < 0.01) (Fig. 3a,b). The transcripts of Sterol regulatory element binding protein 1 (SREBP-1) and Fatty acid synthetase (FAS) were lower in mice fed with diet of 3.8% blended oil than in mice fed with diet containing 3.8% lard or soybean oil (*P* > 0.05). However, there was dramatic reduction in BO-H while compared with SO-H (*P* < 0.01) (Fig. 3c,d). The expression of SREBP-1 in BO-H was significant lower than LO-H (*P* < 0.05) (Fig. 3c). Adipose triglyceride lipase (ATGL) and Adiponectin (APN), indicators of lipolysis, were significantly diminished in SO-H whereas its content was higher in LO-Hand BO-H (*P* < 0.01), (Fig. 3e,f). Leptin, which assists to reduce fat mass, was remarkably decreased in BO-H while compared to LO-H and SO-H (*P* < 0.01) (Fig. 3g). We finally measured the protein levels of mice fed with different oils. As shown in Fig. 4b–d, the levels of PPARγ, SREBP1 and FAS were changed by blended oil, which was consistent with the data of transcripts by real time PCR. In addition, blended oil significantly up regulated ATGL protein level while compared with mice fed with soybean oil and lard (*P* < 0.01), and soybean oil led to the lowest ATGL level (Fig. 4e). These results implicate an effective protection of blended oil against adipose cell differentiation and synthesis, which is in favor of lipolysis.

## Discussion

It has been shown that digestibility is closely associated with contents of oil. In general, there are two factors that have an influence on lipid digestibility, and the first element is the amount of oil intake. It has been suggested that the level of oil in diet is related to lipid digestibility^17,18^. The second element is the chain length of fatty acid and the proportion of SFAs, MUFAs and PUFAs. It has been reported that soybean oil, which has high polyunsaturated fatty acids, enhances nutrient digestibility^19^. This finding has been verified in this study showing that the weight and body fat rate of mice fed with diet 6.5% soybean oil increased, which may be associated with the increased percentage of UFAs of FAs, particularly the amount of PUFAs (nearly 59%), and its proportion was significantly higher than lard and soybean oil.

Cholesterol, an important lipid, comprises the structure of cell membrane. Low-density lipoprotein (LDL) is responsible of transferring endogenous cholesterol from liver to peripheral tissues. The accumulation of cholesterol in serum is strongly related to the increase of LDL-C in serum^20^. High-density lipoprotein (HDL) is a lipoprotein which plays a role in anti-atherogenic property by reversing cholesterol transport from the peripheral tissues to liver. The content of HDL-C, LDL-C and TG was increased in mice fed with 6.5% blended oil compared to 6.5% lard and soybean oil. This is likely to be attributed to the effect of the increased metabolism of lipid in liver and adipose. Additionally, the ratio of serum HDL-C and LDL-C is one of the most intuitive evaluation indicators for serum lipids. In mice fed with 3.8% blended oil, the ratio of HDL-C/LDL-C is lower than the other groups. It is obvious that those levels of blood lipids can be adjusted with diet oil blended with lard and soybean at 25 g per day for one person.

When the lipid transfer or metabolism in the liver exceeds the range of its own capacity, it causes too much ectopic fat deposition which results in nonalcoholic fatty liver disease^21^. We found that there was obvious decrement of TG in liver in mice fed with diet 6.5% oil. In liver sections, lipid deposition was not observed. In contrary to TG in liver, TG in fat was significantly increased in mice fed with diet 6.5% oil. It was suggested that TG was mainly transported to adipose tissue for storage while taking 6.5% oil. The mechanism underlying the distinction of lipid transport between mice fed with diets of different proportion of oils needs to be explored further.

Adipose tissue plays a crucial role in lipid storage, energy homeostasis, and insulin sensitivity in the body. The redundant energy uptake from food is stored in adipose tissues in the form of triglyceride, and released to plasma in the form of fatty acid. The redundancy of subcutaneous and visceral adipose are the main factors of obesity. Adipogenesis is the process of cell differentiation by which pre-adipocytes become mature adipocytes in adipose tissues, and the morphology and function of cells were changed from pre-adipocytes to mature adipocytes.

It has been demonstrated that the level of PPARγ expression is increased during the development of readipocytes into mature adipocytes^22^, and that the differentiation process could not complete with the deficiency of PPARγ^23^. C/EBPα is also important for adipogenesis, and C/EBPα knockout mice fail to accumulate lipids in adipose tissues, and die of hypoglycemia and liver defect shortly after birth. In this study, the mRNA and protein expression levels of PPARγ were higher in the adipose of mice fed with 6.5% lard and 6.5% soybean oil or blended oil, and their transcripts were higher in mice fed with 6.5% lard than fed 3.8% lard. The C/EBPα mRNA expression level also higher in the adipose of mice fed with 6.5% lard than the mice fed with the same volumes of the other two types of oils. Previous studies have reported that the natural ligands of PPARs mainly include natural or modified PUFAs and eicosanoid^24,25^, and the inhibiting adipogenesis effect of the SFAs-derived feeds are stronger than the UFAs-derived diets in swine^26^. Our results revealed that the mRNA expression levels of PPARγ and C/EBPα were parallel to the volume of lard and SFAs, which is inconsistent with the studies showed above. Duan *et al*. reported that a diet with a lower n-6: n-3 PUFA ratio could reduce the expression levels of PPARγ in pig models^27^, we speculated that the lower expression levels of PPARγ and C/EBPα on account of a lower n-6: n-3 PUFA ratio in blended oil, which need to be further examined. Collectively, the higher doses of lard, improves the differentiation of adipocytes in adipose tissues of mice.

In mammals, fatty acids are necessary for lipid accumulation, and the principle source of fatty acids is *de novo* synthesis in liver and adipose tissues. FAS is the key enzyme of *de novo* synthesis for endogenous fatty acids, which is mainly expressed in liver and adipose tissues and catalyze carbohydrates to fatty acids. SREBP-1 belongs to the family member of transcription factors that regulate cellular lipogenesis and lipid homeostasis and control, it regulates the expression of several enzymes that are involved in the synthesis of cholesterol, fatty acid, triacylglycerol and phospholipid^28^, which increases the expression of FAS and PPARγ mRNA and participates in the formation of endogenous ligands for PPARγ^29^. In this study, both of the SREBP1 and FAS mRNA expression levels in adipose of mice fed with 3.8% oils were lower than the mice fed with 6.5% oils. Among the mice fed with 6.5% oils, the mRNA and protein expression levels of SREBP1 and FAS in adipose of mice fed with soybean oil were higher than the mice fed with lard and blended oil. Previous researchers have showed that PUFAs have remarkable effects on reducing expression of FAS mRNA, but increasing the level of SFAs^30,31^. From the perspective of fatty acids of oils, we found the contrary results, showing that high dose of soybean oil, which is rich in PUFAs, has the ability to stimulate the expression levels of SREBP-1 and FAS. Considered together, digestibility for lipids and other nutrients is stronger in mice fed with 6.5% soybean oil than mice fed with lard and blended oil. Higher dose of soybean oil has the potential to improve lipogenesis in adipose of mice. However, higher concentration of blended oil may have the reverse effect.

ATGL plays an essential role in lipolysis—a series of orderly controlled process of TG hydrolysis, which leads to the formation of DG and FA^32^. In contrast to wild type mice, there is a reduction of FA release from WAT by more than 75% in ATGL-deficient mice^33^. We observed that higher lard enhanced the expression level of ATGL mRNA, which was in accordance with the expression of PPARγ mRNA and protein. This was consistent with previous studies showing that the expression of ATGL mRNA is positively regulated by PPARγ^34,35^. APN is a proteohormone that is secreted by adipose, and the circulating APN is involved in appetite, glycometabolism and lipid metabolism regulation. Leptin has been regarded as a signal in the brain to inhibit food intake and decrease weight^36^. The secretion concentrations of both leptin and APN showed positive correlation with mRNA expression levels. Obesity is accompanied by high leptin levels and leptin resistance^37^, and consequently the circulating leptin level is proportional to adipose tissue mass^38^. Among the mice fed with 6.5%t oils, lard had a distinct ability to increase the expression level of APN mRNA, but soybean oil assumed a reverse effect. The expression levels of APN mRNA were also in accord with PPARγ mRNA, which is confirms with the previous findings reported by Li *et al*.^39^. In the present study, a lower expression level of leptin mRNA in mice fed with blended oil at 6.5% level, suggesting that a lower n-6: n-3 PUFA ratio in blended oil, which is agreement with previous findings^40^. The expression levels of leptin mRNA approximately identify with body fat rate in mice, indicating that leptin resistance occurs in the obese mice fed with higher soybean oil. Therefore, 6.5% lard or blended oil has a much stronger capacity of stimulating lipolysis than soybean oil in mice.

In summary, we have demonstrated that the blended oil has a significant effect on anti-obesity. The blended oil also has the ability of decreasing blood lipids, preventing the damage of lipid peroxidation in liver and reducing the abnormity of renal function. The mechanisms of anti-obesity effect of blended oil may be attributed to the regulation of PPARγ, C/EBPα, SREBP1, FAS and ATGL. In adipose tissues, the processes of adipogenesis and lipogenesis are suppressed, and the hydrolysis of stored triglycerides is promoted. As a result, body weight and body fat rate of the subjects are decreased. Soybean oil at the intake of current Chinese residents (41.4 g/d), results in the increase in the body weight and body fat mass. Alternatively, lard at 41.4 g/d leads to the reduction in anti-oxidant in liver, an increase in the burden of kidney, an elevation of adipogenesis and hydrolysis of triglycerides in adipose tissues. We have revealed that the traditional Chinese eating habit, taking in oils blended with lard and soybean oil, is one of the factors of lower percentage of overweight and obesity in China. This study provides a novel insight into reasons for increasing rates of obesity over the past 20 years in China, which might be attributed to the increase of dietary oil intake and the change of the oil components.

## Materials and Methods

### Animals, diets and experimental design

Male C57BL/6 J mice (n = 60, 8 weeks old) were purchased from Hunan Silaike Laboratory Animal Co., Ltd (Changsha, China). Soybean oil was purchased from China Oil & Foodstuffs Co.,Ltd (Beijing, China), and the name of product was Fu Lin Men First Grade Soybean Oil. Lard was obtained from Hunan Yancun Ecological Agriculture & Animal Husbandry Tech Co., Ltd (Changsha, China).

All mice were housed in a room with standard environment and given food and water *ad libitum*. After acclimation for 1 week, the mice were randomly divided into the following 6 groups (n = 10/group): SO-L (3.8% soybean oil of diet), LO-L (3.8% lard of diet), BO-L (3.8% blended oil of diet), SO-H (6.5% soybean oil of diet), LO-H (6.5% lard of diet), BO-H (6.5% blended oil of diet), and composition of the diets was shown in Table S1. The blended oil consisted of 54% soybean oil and 46% lard. The two levels of oils were simulated to the volume of oil intake suggested by China Public Union of Nutrition (25 g per day) and the volume of oil intake practically among Chinese citizen (42 g per day), respectively. Mice were fed by experimental diets for 12 weeks, which was proximately equivalent to 12 years in human. During the feeding period, the mice were weighed every week, and food intake was measured every day in twelfth weeks. At the end of experiment, all mice were fasted for 12 h before they were sacrificed. The mice were sacrificed by cervical dislocation under anesthetized and their blood and organs were collected. All of the procedures were conducted according to the guidelines of the Institutional Animal Care and Use Committee of Hunan Agricultural University and were approved by the Institutional Ethics Committee of Hunan Agricultural University.

### Samples collection and tissues preparation

Blood samples were collected from the retro orbital plexuses, and serum was isolated by centrifugation at 3,900 g for 10 min at 4 °C and immediately stored at −80 °C. The liver and adipose (epididymal, mesenteric and perirenal adipose tissues) of each mouse were harvested, weighed and taken photograph, respectively. Left lobe of livers and one of epididymal adipose tissues were fixed in 10% neutral buffered formalin, and the rest of livers and epididymal adipose tissues were cut into 6 pieces, washed with saline, and frozen immediately at −80 °C.

### Serum biochemical parameters

The content of TG, LDL-C, HDL-C, were determined using Mindray Biochemical analyzer BS-190 (Mindray, Shenzhen, China).

### Histopathological examination

Left lobe of livers and epididymal adipose tissues were fixed in 10% neutral buffered formalin for more than 24 h, and they were embedded in paraffin block. In general, 4-5 µm sections were cut and mounted on glass slides. Paraffin was removed with xylen and alcohol. The sections were stained with hematoxylin and eosin (H&E). After dehydration by alcohol and mounting with neutral balsam, the photographs were taken with Olympus photomicroscope (Olympus Inc., Tokyo, Japan). Adipocyte area of H&E-stained cross-sections was measured in the 5 fields of 5 individual samples in each group, and calculated by Image-Pro Plus 5.1 (Media Cybernetics, Inc. Silver Spring, Maryland, USA).

### Lipids in liver and adipose tissue and oxidative stress in liver

The frozen livers were homogenized in saline and used to examine lipids content. Total proteins in the tissue homogenate were determined by the Coomassie brilliant blue method using a total protein assay kit. TG and TC levels were determined by GPO-PAP method and COD-PAP method, respectively. The frozen epididymal adipose tissues were homogenized in alcohol, and TG and TC contents of the homogenate were determined by GPO-PAP and COD-PAP method. All of these assay kits were purchased from Nanjing Jiancheng Bioengineering Institute (Nanjing, China).

### RNA isolation and quantitative real time-PCR (qRT-PCR)

Total RNAs were isolated from frozen epididymal adipose tissues using the TRIzol reagent (Invitrogen, Carlsbad, USA) according to the manufacturer’s protocols of the manufacturer. The mRNAs were reverse transcribed to cDNAs using the PrimeScript RT reagent Kit (Takara Bio Inc., Japan), according to the manufacturer’s instructions. qRT-PCR was performed using the StepOne Real-Time PCR System (Applied Biosystems, USA) with the SYBR Premix Ex Taq II Kit (Takara Bio Inc., Japan), according to the protocols.

Briefly, PCR was performed in a reaction volume of 20 μL per well, containing 2 μL (200 ng) of cDNA, 0.8 μL of 10 μmol/L forward and reverse primers, 10 μL of SYBR Premix Ex Taq II, 0.4 μL of ROX Reference Dye, and 6 μL deionized water. PCR reactions started by an initial denaturing step at 95 °C for 30 s, followed by 40 cycles of 5 s at 95 °C and 30 s at 60 °C. Primers of chosen genes were listed in Table S1. Relative CT amounts were normalized to β-actin expression, and the results are calculated using the 2^−ΔCT^ method.

### Western blots

Frozen epididymal adipose tissues were lysed in RIPA buffer (Beijing Solarbio Science & Technology Co., Ltd., Beijing China) at 4 °C. The lysates were centrifuged at 18,200 g for 10 min at 4 °C, and the supernatant was collected. The total protein content in the lysate was measured by the BCA method using a protein assay kit (Nanjing Jiancheng Bioengineering Institute, Nanjing, China). Equal amounts of proteins were electrophoretically separated by 15% sodium dodecyl sulphate-polyacrylamide gel (SDS-PAGE) and then transferred onto polyvinylidene difluoride (PVDF) membranes. The membranes were blocked in 5% non-fat dry milk-TBST buffer for 1 h at room temperature. After being rinsed, the membranes were incubated overnight at 4 °C with the primary antibodies, including SREBP-1 (BioSS Biotechnology Co., Ltd., China), PPARγ (Santa Cruz Biotechnology, Inc., USA), FAS (abclonal Technology Co., Ltd., China), ATGL(Cell Signaling Technology, Inc. USA), and GAPDH(Wuhan goodbio technology CO.,LTD., China). The membranes were next incubated with horseradish peroxidase conjugated-secondary antibody (Wuhan goodbio technology CO.,LTD., China) for 1 h and washed with TBST buffer. Bands were visualized with an enhanced chemiluminescence kit (Nanjing KeyGen Biotech. Co. Ltd., China), and the intensities of the bands were quantified by AlphaEase FC software (Alpha Innotech Co., USA).

### Statistical analysis

All results were presented as means ± s.e.m. Comparisons between groups were performed using one-way ANOVA with the LSD post hoc analysis. Differences were considered to be statistically significant when P < 0.05. Statistical analysis was performed with SPSS 17.0 (SPSS Inc., Chicago, USA), and the bar graphs of data were made by GraphPad Prism version 7 (GraphPad Software, San Diego, USA).

## Electronic supplementary material

Supplementary Tables
