# Supplementary material for: Anti-obesity effect of a traditional Chinese dietary habit—blending lard with vegetable oil while cooking
Source: Sci Rep. 2017 Oct 31;7:14689. doi: 10.1038/s41598-017-14704-2 (PMC5665938; doi:10.1038/s41598-017-14704-2)
Supplement: Supplementary file 1 — Supplementary Tables [file 41598_2017_14704_MOESM1_ESM.pdf]

Anti-obesity effect of a traditional Chinese dietary habit—blending lard with vegetable oil while cooking.

Ji Wang[1,+], Sisi Yan[1,+], Haisi Xiao[1], Huijuan Zhou[1], Shuiping Liu[1], Yu Zeng[1], Biying Liu[1], Rongfang Li[1], Zhihang Yuan[1], Jing Wu[1], Jine Yi[1], Yarou Bao Sero Razack[1] & Lixin Wen[1,2,*]

[1]Laboratory of Animal Clinical Toxicology, Department of Clinical Veterinary Medicine, College of Veterinary Medicine, Hunan Agricultural University, Changsha, Hunan Province, P.R. China.

[2]Hunan Collaborative Innovation Center of Animal Production Safety, Changsha, Hunan Province, P.R. China.

[+]These authors contributed equally to this work.

[*]Correspondence and requests for materials should be addressed to Lixin Wen (E-mail: sfwlx8015@sina.com).

Supplementary Tables

Table S1. Composition of the diets (g/kg)

**Ingredient**[1]	**LSO**	**LLO**	**LBO**	**HSO**	**HLO**	**HBO**
Corn starch	550	550	550	550	550	550
Wheat bran	102	102	102	65	65	65
Soybean meal	170	170	170	180	180	180
Fish meal	80	80	80	80	80	80
Beer yeast	20	20	20	20	20	20
Soybean oil	38	0	20.5	65	0	35.1
Lard oil	0	38	17.5	0	65	29.9
Premix	40	40	40	40	40	40
Total energy (kcal/kg)[3]	3450	3450	3450	3610	3610	3610
Energy from lipid	20%	20%	20%	25%	25%	25%

[1]. The ingredients of diets were prepared according to Laboratory Animals Nutrients for Formula Feeds *GB 14924.3-2010*.

[2]. Premix was mixed with vitamins, amino acids and minerals according *GB 14924.3-2010*.

[3]. Energy from carbohydrate and protein was 4 kcal/g. Energy from lipid was 9 kcal/g.

Table S2. Primer sequences of genes used for quantitative real-timePCR

Genes	GenBank accession	Primer sequences	PCR size (bp)
β-actin	NM_007393	F: CATCCGTAAAGACCTCTATGCCAAC<br>R: ATGGAGCCACCGATCCACA	171
SREBP-1	NM_011480	F: GGAGGCAGAGAGCAGAGATG<br>R: CACAGGTTCCCCATAGACAAA	124
PPARγ	NM_001127330	F: TCGCTGATGCACTGCCTATG<br>R: GAGAGGTCCACAGAGCTGATT	103
C/EBP-α	NM_007678	F: CAGGGCAGGAGGAAGATACA<br>R: AGACTCAAATCCCCAACACCT	107
FAS	NM_007988	F: CTGTCTGGGCATAACGGTCT<br>R: GCGGTGTGAAAACGAACTTT	119
ATGL	NM_001163689	F: CAACGCCACTCACATCTACGG<br>R: GGACACCTCAATAATGTTGGCAC	106
Leptin	NM_008493	F: TTCACACACGCAGTCGGTATC<br>R: GGCTGGTGAGGACCTGTTG	131
APN	NM_009605	F: TGTTCCTCTTAATCCTGCCCA<br>R: CCAACCTGCACAAGTTCCCTT	104
